# A novel technique for determining the axis of the fetal heart: Clock position method

**DOI:** 10.4274/jtgga.galenos.2020.2019.0177

**Published:** 2020-09-03

**Authors:** Sezgin Dursun, Fatih Aktoz

**Affiliations:** 1 Private Clinic, Ağrı, Turkey; 2 Clinic of Obstetrics and Gynecology, Ağrı State Hospital, Ağrı, Turkey

To the Editor,

Determination of the fetal heart axis via ultrasonography is crucial as some cardiovascular diseases can only be diagnosed according to the accuracy of fetal heart position. There are reliable methods for ultrasonographic assessment of the left/right axis by using fetal stomach or gallbladder as a landmark. However, if there is a malposition of an indicator organ, it may be difficult to distinguish the left side from the right side. For this reason, these methods sometimes require more experienced physicians to obtain reliable results. For inexperienced clinicians this can be more difficult because both understanding the position of fetus and ultrasound probe orientation can be confusing. There is a need for a practical technique that can be used easily and reliably by perinatologists or obstetricians.

We suggest an easy method for assessment of the fetal heart axis. In our method, the physician should be seated on the right side of the patient, hold the probe with the right hand and perform a transabdominal examination. The left side of the probe should be on the left side of the ultrasound screen. The fetus should be scanned in a transverse plane. The thoracic cavity is imagined as a watch dial and the fetal spine refers the 12 o’clock position. If the fetus is in breech presentation, the axis of the heart should be approximately at the 7 o’clock position (Figure 1a) and similarly, if the fetus is in cephalic presentation, the axis of the heart should be approximately at the 5 o’clock position (Figure 1b). If the fetus is in a transverse lie, indicator of the probe should point to patient’s head for sagittal imaging and the fetal structure nearest to clinician (in other words closest part of the fetus to maternal right side) is accepted as the presenting part.

Other methods have been described to assess of the fetal heart position. Cordes et al. (1) described a technique in 1994. The left and right side of the fetus was distinguished depending on parameters such as position of fetus and mother, ultrasound probe orientation and video screen. Although being accurate and reliable, it is not easy to learn and perform for all practitioners. Bronshtein et al. (2) suggested a simple technique to determine fetal situs. They used forearm, hand and thumb for orientation. In this method, right hand for transabdominal examination and left hand for transvaginal examination was used. The dorsal side of the forearm referred to the fetal back and thumb showed the fetal left side. It is more user-friendly than Cordes et al.’s (1) technique but clinicians can be confused when they choose the appropriate hand to use for evaluation. The method that we described can be used by any clinician. It does not require calculation. Thus, it requires less time compared to others. In addition, our method is not affected by fetal movements. In conclusion, we believe that this technique may be a good option for clinicians at every level of experience.

## Figures and Tables

**Figure 1:**
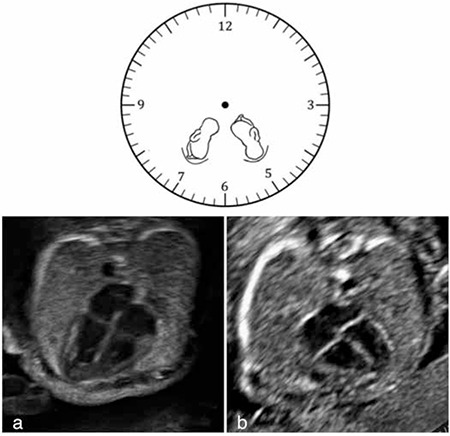
Clock position method to determine fetal heart axis. The spine always refers to the 12 o’clock position (a) Breech presentation; the axis of the heart at the 7 o’clock position. (b) Cephalic presentation; the axis of the heart at the 5 o’clock position
